# Harnessing Social Media Data to Understand Information Needs About Kidney Diseases and Emotional Experiences With Disease Management: Topic and Sentiment Analysis

**DOI:** 10.2196/64838

**Published:** 2025-02-25

**Authors:** Hee Jeong Hwang, Nara Kim, Jeong Yun You, Hye Ri Ryu, Seo-Young Kim, Jung Han Yoon Park, Ki Won Lee

**Affiliations:** 1 Department of Agricultural Biotechnology College of Agriculture and Life Sciences Seoul National University Seoul Republic of Korea; 2 Advanced Institutes of Convergence Technology Seoul National University Suwon Republic of Korea; 3 Bio-MAX Institute Seoul National University Seoul Republic of Korea; 4 Institutes of Green Bio Science & Technology Seoul National University Pyeongchang Republic of Korea; 5 Department of Agricultural Biotechnology and Center for Food and Bioconvergence Seoul National University Seoul Republic of Korea

**Keywords:** kidney diseases, online health communities, topic modeling, sentiment analysis, disease management, patient support

## Abstract

**Background:**

Kidney diseases encompass a variety of conditions, including chronic kidney disease, acute kidney injury, glomerulonephritis, and polycystic kidney disease. These diseases significantly impact patients’ quality of life and health care costs, often necessitating substantial lifestyle changes, especially regarding dietary management. However, patients frequently receive ambiguous or conflicting dietary advice from health care providers, leading them to seek information and support from online health communities.

**Objective:**

This study aimed to analyze social media data to better understand the experiences, challenges, and concerns of patients with kidney disease and their caregivers in South Korea. Specifically, it explored how online communities assist in disease management and examined the sentiment surrounding dietary management.

**Methods:**

Data were collected from *KidneyCafe*, a prominent South Korean online community for patients with kidney disease hosted on the Naver platform. A total of 124,211 posts from 10 disease-specific boards were analyzed using latent Dirichlet allocation for topic modeling and Bidirectional Encoder Representations From Transformers–based sentiment analysis. In addition, Efficiently Learning an Encoder That Classifies Token Replacements Accurately–based classification was used to further analyze posts related to disease management.

**Results:**

The analysis identified 6 main topics within the community: *family health and support*, *medication and side effects*, *examination and diagnosis*, *disease management*, *surgery for dialysis*, and *costs and insurance*. Sentiment analysis revealed that posts related to the *medication and side effects* and *surgery for dialysis* topics predominantly expressed negative sentiments. Both significant negative sentiments concerning worries about kidney transplantation among family members and positive sentiments regarding physical improvements after transplantation were expressed in posts about family health and support. For *disease management*, 7 key subtopics were identified, with inquiries about dietary management being the leading subtopic.

**Conclusions:**

The findings highlight the critical role of online communities in providing support and information for patients with kidney disease and their caregivers. The insights gained from this study can inform health care providers, policy makers, and support organizations to better address the needs of patients with kidney disease, particularly in areas related to dietary management and emotional support.

## Introduction

### Background

Kidney diseases primarily manifest in 2 main forms: chronic kidney disease (CKD) and acute kidney injury (AKI), which are 2 significant kidney conditions [1,2]. CKD is characterized by a progressive decline in kidney function lasting >3 months, whereas AKI refers to a sudden decrease in kidney function that occurs over a period of hours to days [1,2]. Both conditions can arise from various underlying causes, including primary glomerular diseases (eg, glomerulonephritis), inherited disorders (eg, polycystic kidney disease), and secondary complications of systemic conditions such as diabetes and hypertension [2,3]. These conditions collectively affect a significant proportion of the global population, with CKD alone estimated to affect 13.4% of people worldwide [4]. Both CKD and AKI, regardless of their underlying etiology, increase the risk of other medical complications and significantly impact patients’ quality of life and health care costs [2,5]. Managing these conditions can require substantial lifestyle changes, particularly in dietary habits, which can be challenging for patients [6]. However, maintaining a healthy lifestyle can reduce the risk of kidney disease progression and lower mortality rates [7].

Despite the critical role of lifestyle modifications in managing kidney diseases, patients often report receiving ambiguous or conflicting information from health care providers, leading to confusion and a sense of disempowerment [8]. Time constraints in clinical settings further limit the extent of guidance that health care providers can offer, leading many patients to turn to online health communities for information and support.

The use of web-based platforms for health-related information has become increasingly prevalent, with approximately 80% of patients using the internet to exchange information and over one-third of adults using social media for health information and social support [9,10]. Unlike traditional research methods such as surveys or interviews, social media analysis provides access to authentic, unprompted patient discussions, offering unique insights into their daily challenges and evolving needs [9,10]. This approach has been widely used to understand patient experiences within chronic disease communities. For example, studies have used social media analysis to capture patient narratives about cancer [11] and examine the social media behaviors of patients and health care providers across various conditions, including asthma, mental health disorders, diabetes, and gout [12,13].

While topic modeling has been widely used to extract health-related topics from social media, studies specifically examining public discourse on kidney diseases are scarce, particularly in South Korea. Despite the country’s high internet penetration and active online health communities, research that systematically analyzes patient experiences with kidney disease through social media remains limited. Successful applications of social media analysis in South Korea include studies on diabetes-related communication on social networking services [14], evaluations of physician-created thyroid cancer content on YouTube [15], the use of topic modeling for pancreatic cancer information [16], and investigations into cross-cultural perceptions of urological conditions [17]. However, comprehensive studies of kidney disease communities are notably absent. These communities often reflect unique cultural challenges and interactions with the health care system that are specific to South Korean patients.

Recent advances in natural language processing (NLP) have transformed how researchers analyze and interpret unstructured health-related text data. Deep learning approaches in NLP have shown significant success in processing various types of health care text, including clinical narratives, electronic health records, and social media content [18,19]. These computational methods are particularly effective in uncovering patterns and insights that are not readily apparent through traditional analytical methods. Applications of NLP in health care range from extracting medical entities and relationships from clinical texts to analyzing patient sentiments, behaviors, and experiences shared in online health communities. Such approaches are instrumental in bridging the gap between raw data and actionable insights in health care research.

While topic modeling allows for quantitative analysis of discussed topics, it does not fully capture the emotional and experiential dimensions of patients’ lives. To address this limitation, our study combined topic modeling with sentiment analysis, using advanced NLP techniques to provide both quantitative insights and a qualitative understanding of patients’ emotional challenges. This dual approach bridges the gap between unstructured patient narratives and actionable health care insights. By focusing on the experiences of South Korean patients with kidney disease, our findings can inform the development of culturally appropriate, patient-centered interventions, enabling health care providers to address patient concerns more effectively, design impactful educational materials, and enhance support systems tailored to these patients’ unique needs.

### Objectives

The main objectives of this study were to (1) analyze social media data using topic modeling to better understand the experiences, challenges, and concerns of patients living with various kidney diseases and their caregivers in South Korea; (2) explore the ways in which online communities can provide assistance, specifically in relation to disease management for different kidney conditions; and (3) examine the sentiment surrounding disease management among individuals with kidney diseases.

By achieving these objectives, this study aimed to provide valuable insights that can inform health care providers, policy makers, and support organizations in better addressing the needs of patients with a spectrum of kidney diseases and renal conditions.

## Methods

### Overview

**Figure 1:**
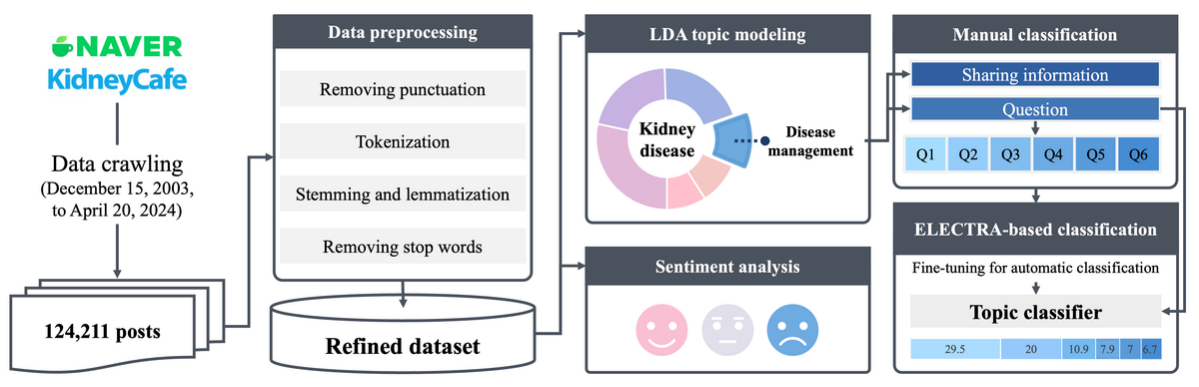
Overall procedures used in the study. The workflow included data crawling from the Naver KidneyCafe, data preprocessing, latent Dirichlet allocation (LDA) topic modeling, sentiment analysis of the total posts, and manual and Efficiently Learning an Encoder That Classifies Token Replacements Accurately (ELECTRA)–based classification related to the disease management topic. Q1: the safety of specific foods; Q2: dietary management strategies; Q3: dietary recommendations; Q4: water intake management; Q5: weight management; Q6: exercise management.

The goals of this study were to identify the challenges faced by patients and determine the key topics related to kidney disease management. We used topic modeling for quantitative analysis and sentiment analysis for qualitative analysis to achieve this. Relevant posts were gathered from online communities discussing kidney disease, preprocessing was conducted, and the topics and sentiments were analyzed. Figure 1 provides an overview of the research methodology.

### Data Collection and Preprocessing

We collected data from *KidneyCafe*, a prominent South Korean online community for patients with kidney disease and caregivers hosted on the Naver platform [20]. This community has approximately 183,545 members and facilitates discussions across various themed boards, each concentrating on specific kidney-related topics. For our analysis, we focused on the disease-specific boards within the *Member Participation* section, which is dedicated to active discussions among engaged community members. These boards address topics such as kidney transplantation, dialysis, and related medical conditions, enabling us to capture in-depth conversations pertinent to a wide array of kidney disease experiences and management strategies.

The Selenium WebDriver was used to extract 124,211 posts from 10 disease-specific boards, dating from the community’s inception on December 15, 2003, to the date of data collection (April 20, 2024). The collected data included post titles and content.

Data preprocessing was conducted using Python (Python Software Foundation), with the *KoNLPy* library used for Korean NLP [21]. This step involved removing punctuation and noninformative words as well as extracting nouns from the text to prepare for subsequent analysis. To handle informal language patterns in user-generated content, we used a custom-built stop word list to exclude frequently appearing, noninformative terms that do not contribute to semantic analysis. This stop word list was created by reviewing existing lists from similar Korean health-related studies [22] and further customizing it to focus on discussions surrounding kidney disease. These preprocessing steps were essential in ensuring the robustness of our analysis and the interpretability of the results.

### Topic Analysis Using Latent Dirichlet Allocation

We used latent Dirichlet allocation (LDA) topic modeling [23], an unsupervised machine learning model, to uncover latent topics in the collected posts. Topic modeling is a technique widely used in NLP for discovering topics and extracting meaning from large, unstructured documents. LDA is the most popular topic modeling method and is based on statistical probability inference. It assumes that each document can be represented by a probabilistic distribution over latent topics, and each topic is characterized by a probabilistic distribution over the words [24].

We imported LDA models from the *Gensim* library for this study. When analyzing topics using LDA, the number of topics is typically determined using coherence scores such as U_mass and perplexity [25,26]. The U_mass coherence score, which is based on word co-occurrence statistics within documents, has been validated to closely correspond with human labeling results [27,28]. This score has been effectively used in analyzing health-related social media topics, including obesity, COVID-19, and vaccines [27-30]. Similarly, perplexity has proven to be a valuable metric in recent topic modeling studies [31,32]. While a higher coherence score typically indicates better topic quality and interpretability, recent research shows that statistical optimization alone may not guarantee optimal interpretability of topics [32-34]. To tackle this issue, several studies have adopted validation methods that integrate statistical metrics with qualitative assessments. For instance, Min et al [31] combined perplexity and coherence metrics with qualitative evaluations in their analysis of occupational accidents, whereas Lyu et al [33] and Li et al [32] emphasized the significance of semantic coherence alongside statistical validation.

**Figure 2:**
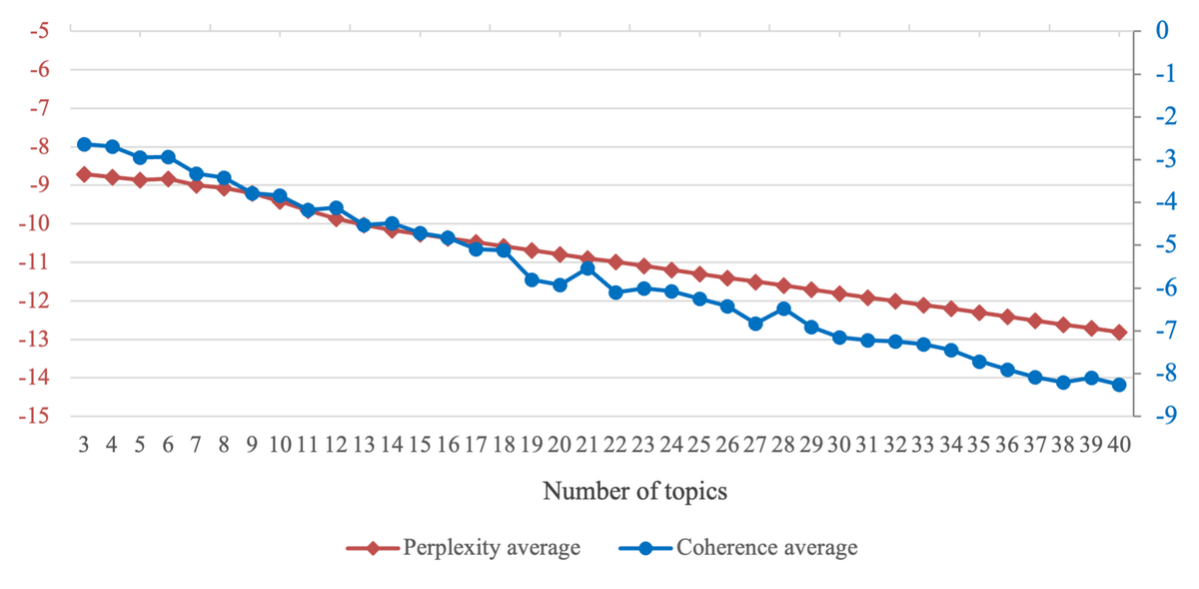
Perplexity and U_mass coherence scores across different numbers of topics. This figure shows the perplexity and U_mass coherence scores as the number of topics varies from 3 to 40 using 10-fold cross-validation. Both metrics indicate better performance with a lower number of topics.

**Figure 3:**
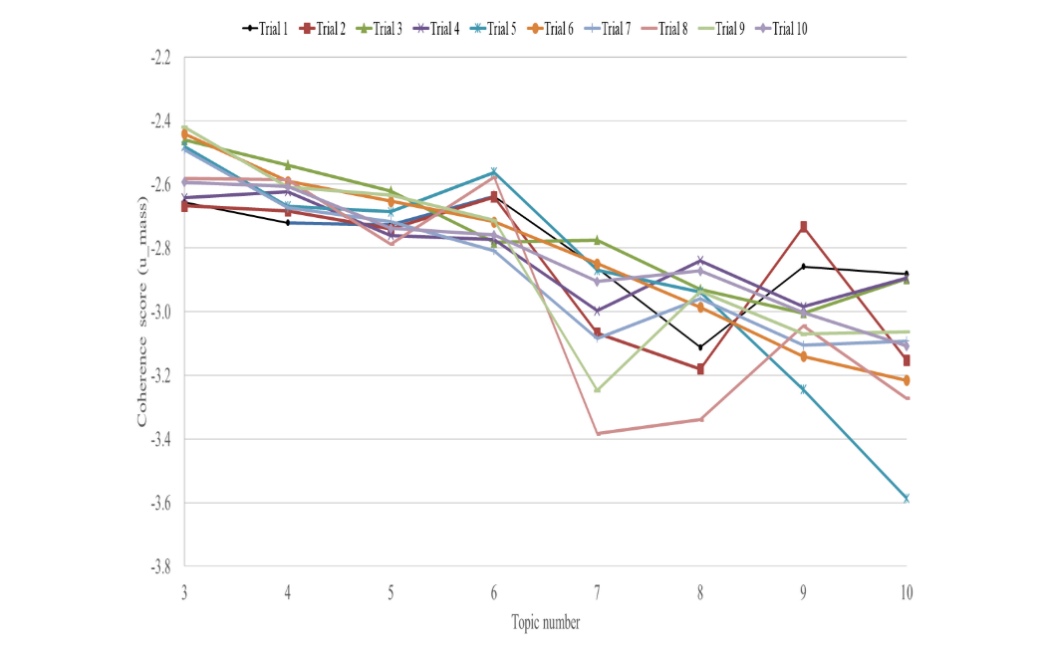
U_mass coherence scores for 3 to 10 topic clusters across 10 trials. This figure shows the U_mass coherence scores as the number of topic clusters increases from 3 to 10 across 10 separate trials. The highest coherence score is observed when there are 3 and 6 topic clusters.

Following established approaches [32-34], we first calculated perplexity and U_mass coherence scores across a range of topics (3-40) using 10-fold cross-validation. As shown in Figure 2, both metrics demonstrated better performance with fewer topics, leading us to concentrate on the range of 3 to 10 topics. Within this range, we conducted 10 iterations of the model to ensure consistency and, ultimately, selected 6 topics. This decision was based on a combination of qualitative assessments of topic interpretability and the high U_mass coherence scores observed at both 3 and 6 topics (Figure 3).

Using the optimal cluster size, we ran the LDA model to obtain lists of representative words for each cluster. The topic-labeling process involved 4 researchers, each with diverse expertise in nutrition and food science, who collaboratively reviewed and interpreted the word lists. We followed established methodologies in social media analysis [33,35,36] using an iterative, discussion-based approach. Initially, each researcher independently analyzed the word lists generated by the topic modeling algorithm, focusing on the most significant terms and their relative weights within each topic to identify potential themes. Next, they reviewed representative posts selected for their strong association with each topic to contextualize the keywords and understand their relevance to the experiences and concerns of patients with kidney disease. This independent review enabled researchers to form initial interpretations and propose tentative labels for further discussion.

After the independent review, the researchers gathered to compare their initial interpretations and collaboratively refine the proposed labels through discussion. When differing interpretations emerged, the team reviewed additional example posts from the topic clusters to gain more context. This process included examining word clouds and representative posts that distinctly exemplified each topic while also considering the specific experiences and information-seeking behaviors of patients with kidney disease. Final topic labels were assigned once all 4 researchers reached a consensus through comprehensive discussion and contextual analysis.

The contribution of each word to a cluster was evaluated using the probability metric derived from the model results. Representative example sentences for each cluster were identified based on the probability that each post belonged to the topic.

### Sentiment Analysis

The Bidirectional Encoder Representations From Transformers (BERT) method, a machine learning technique for NLP, was used to identify the sentiment of posts about dietary management—positive, negative, and neutral—to uncover which aspects of dietary management were the most challenging. While word embeddings can be derived from large and unannotated corpora using word co-occurrence statistics, they do not consider the context when creating word vectors [37]. However, BERT is context dependent; it produces dynamic and context-specific representations of the word by accounting for neighboring words, which helps interpret the semantics along with the grammatical structure [38].

The sentiment analysis in this study used the Naver CLOVA Sentiment platform, which uses a pretrained BERT model specifically adapted for analyzing Korean-language texts. This adaptation enables the model to effectively capture context-specific nuances, particularly in the informal and colloquial language common in social media discussions. While the specific parameters and fine-tuning details of the application programming interfaces are proprietary to Naver and cannot be disclosed, the effectiveness of this methodology for Korean sentiment analysis has been validated through several academic studies [39-41]. The platform provides sentiment classification results (ie, which sentiment—positive, negative, or neutral—is best represented in the texts), along with the expected probability of each sentiment.

### Analysis of the Disease Management Topic Using Efficiently Learning an Encoder That Classifies Token Replacements Accurately

The *disease management* topic was investigated by conducting a detailed analysis. One author manually classified the posts categorized under *disease management* by the LDA results into questions and nonquestions. In total, 3 additional authors independently annotated a subset of 300 posts to establish the annotation rules. The posts were reviewed for seven key sentiments: (1) general dietary management methods, (2) food recommendations, (3) inquiries about the safety of consuming specific foods, (4) requests for dietary evaluations, (5) water management, (6) exercise management, and (7) weight management. After discussion, the annotators agreed on a finalized set of annotation rules and guidelines.

Next, 2 independent annotators labeled a random set of 1000 posts from the overall dataset. The Cohen κ coefficient for interrater reliability was 0.77, indicating substantial agreement. These annotated data were used for transfer learning. We fine-tuned the Efficiently Learning an Encoder That Classifies Token Replacements Accurately (ELECTRA) model, a pretrained language model that has demonstrated state-of-the-art performance across various Korean-language tasks [42]. Compared to BERT and the A Robustly Optimized BERT Pretraining Approach model, ELECTRA offers both computational efficiency and improved performance on downstream classification tasks [43], making it particularly suitable for handling large-scale datasets such as ours. In addition, ELECTRA’s generator-discriminator training mechanism has been shown to outperform masked language models in tasks that require nuanced contextual understanding, as validated in previous Korean NLP studies [42]. We used 1000 manually annotated posts split into training and test sets at a ratio of 80:20. The evaluation metrics on the test set yielded an *F*_1_-score of 0.85, indicating high performance. This fine-tuned ELECTRA model was then used to annotate the question posts related to the topic of *disease management*.

### Ethical Considerations

This study used preexisting publicly accessible data with no identifiable information and therefore does not require institutional review board review per Federal Regulations for the Protection of Human Research Subjects (45CFR 46.104(d)(4)) or patient consent [44]. In compliance with these guidelines and established research practices, all identifying information, such as usernames and specific identifiers, was anonymized or removed before analysis. This approach is consistent with methodologies used in similar international studies using publicly accessible data [33,45,46], as well as previous research analyzing public Naver Cafe posts [47-49].

## Results

### Community Overview

**Figure 4:**
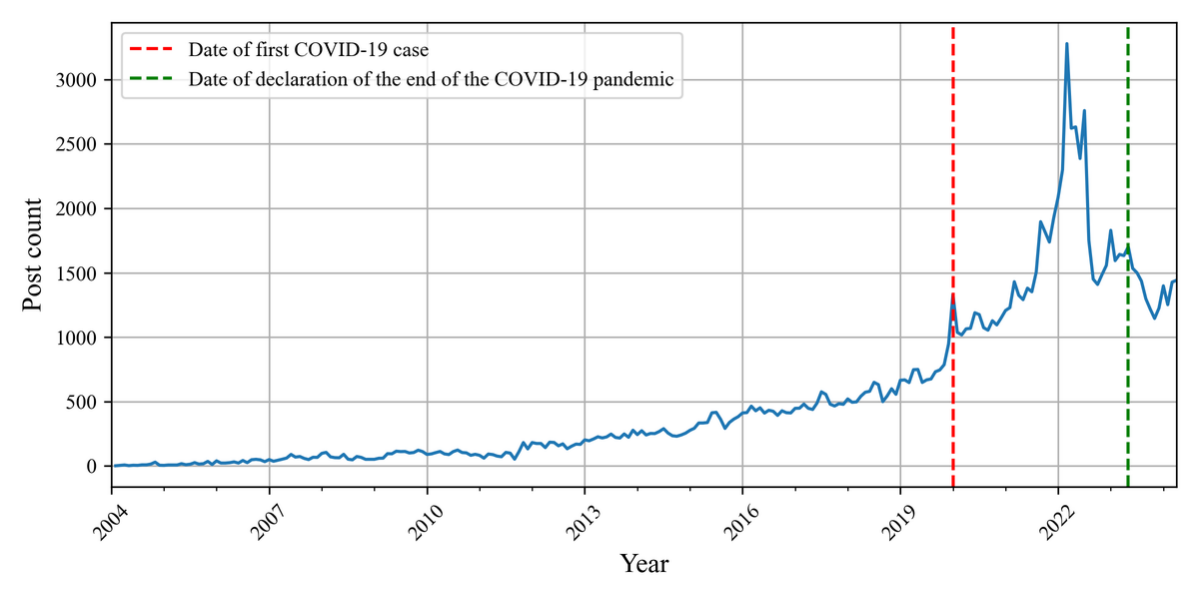
Timeline of the number of posts per month. The red line indicates the first confirmed COVID-19 case in South Korea, whereas the green line marks the World Health Organization’s declaration of the end of the COVID-19 emergency. Monthly post counts were aggregated.

We gathered a comprehensive dataset that included 124,211 posts from various discussion boards in the KidneyCafe community to better understand the experiences, challenges, and concerns of patients living with kidney disease and their caregivers in South Korea. These posts were written by 34,472 unique users, indicating a highly engaged community. Of these users, 34.92% (12,039/34,472) wrote multiple posts, with an average of 3.6 (SD 7.3) posts per user. This high level of repeated engagement suggests that many users found ongoing value and support within the community. Figure 4 illustrates the monthly distribution of posts, showing a significant increase during the beginning of the COVID-19 pandemic in South Korea.

One of the notable advantages of this community is the categorization of discussion boards according to specific diseases. An examination of the number of posts collected per board (Table 1) showed that the highest number of posts was related to kidney transplantation (39,221/124,211, 31.58%), followed by kidney dialysis (27,692/124,211, 22.29%).

**Table 1:** Number of posts per board (N=124,211).

Board^a^	Crawled posts, n (%)
Hypertension & Diabetes	1422 (1.14)
Pediatric Kidney Disease	3271 (2.63)
Others	3969 (3.2)
Polycystic & Cysts & Lumps	4011 (3.23)
Kidney Cancer	6097 (4.91)
Chronic Kidney Failure	8216 (6.61)
Proteinuria & Hematuria	10,113 (8.14)
Nephrotic Syndrome & Glomerulonephritis	20,199 (16.26)
Hemodialysis & Peritoneal Dialysis	27,692 (22.29)
Kidney Transplant	39,221 (31.58)

^a^KidneyCafe organizes posts into boards according to specific health conditions.

### Main Topics Within the KidneyCafe Community

#### Overview

We used LDA for topic modeling to identify the common topics discussed by patients with kidney disease in KidneyCafe. Our analysis revealed 6 distinct topic clusters that represented important discussions within the community. Each cluster was labeled based on the main keywords, as shown in Table 2. The distribution of discussion topics across the community was as follows: 28.36% (35,226/124,211) *family health and support*, 21.11% (26,221/124,211) *medication and side effects*, 20.12% (24,991/124,211) *examination and diagnosis*, 11.67% (14,495/124,211) *disease management*, 9.71% (12,061/124,211) *surgery for dialysis*, and 9.03% (11,216/124,211) *costs and insurance*.

**Table 2:** Keywords representative of the 6 discussion clusters in KidneyCafe.

Topic label	Keywords^a^
Family health and support	“Mother,” “father,” “discharge,” “mind,” “preparation,” “husband,” “start,” “family,” “groom,” “tomorrow,” “sibling,” “call,” “nurse,” “life,” and “body”
Medication and side effects	“Intake,” “immunosuppressant,” “symptoms,” “side effects,” “steroids,” “prescription,” “cold,” “treatment,” “morning,” “rejection reaction,” “dose,” “injection,” “face,” “yesterday,” and “head”
Examination and diagnosis	“Blood pressure,” “tissue examination,” “normal,” “creatinine,” “blood test,” “management,” “creatinine levels,” “outpatient,” “professor,” “maintenance,” “hematuria,” “figures,” “current,” “diagnosis,” and “function”
Disease management	“Exercise,” “water,” “weight,” “food,” “rice,” “meal,” “blood sugar,” “potassium,” “daily,” “diet,” “eat,” “urine volume,” “control,” “diabetes,” and “snack”
Surgery for dialysis	“Blood vessels,” “peritoneal dialysis,” “removal,” “pain,” “recommendation,” “procedure,” “surgical site,” “hemodialysis,” “anesthesia,” “surgery,” “peritoneum,” “machine,” “arteriovenous fistula,” “Seoul,” and “endoscopy”
Costs and insurance	“Application,” “price,” “registration,” “antibodies,” “usage,” “insurance,” “error,” “Korea,” “disease,” “occurrence,” “brain death transplant,” “cross-reaction,” “benefits,” “treatment,” and “donation”

^a^The keywords represent the top 15 terms for each topic ranked by their relevance score from the latent Dirichlet allocation results, making them the most representative words for each topic.

#### Topic 1: Family Health and Support

Topic 1, which accounted for 28.36% (35,226/124,211) of the discussions, mainly focused on family members with kidney disease and concerns regarding their health. The key terms included words related to familial relationships, such as “mother,” “father,” “husband,” “family,” “groom,” and “sibling.” These terms appeared frequently in discussions about kidney transplantation within the family context.

The following is an example of those posts:

It seems like my father will donate a kidney to my mother, but I’m worried.

In addition, concerns and emotions surrounding posttransplant discharge and care were prevalent, with terms such as “discharge,” “mind,” and “preparation” appearing frequently in the posts, as shown as follows:

I work full-time, so how should I take care of my husband after he is discharged?

My sibling offered to donate a kidney, but my mind is very uneasy about it.

#### Topic 2: Medication and Side Effects

Topic 2 comprised 21.11% (26,221/124,211) of the discussions and included posts about the correct use, precautions, and dosages of medications. Key terms such as “intake,” “immunosuppressant,” “prescription,” “treatment,” “morning,” and “dose” were frequently mentioned, highlighting discussions on the proper way to take specific prescription medications. The following are examples of those posts:

I was prescribed Solondo. Can Solondo and immunosuppressants be taken together?

Should immunosuppressants be taken on an empty stomach in the morning?

The community also engaged in discussions on various side effects and their causes, with terms such as “symptoms,” “side effects,” “steroids,” “injections,” and “rejection reaction” being commonly mentioned. The following is an example of such posts:

I was prescribed steroid injections and am experiencing side effects.

#### Topic 3: Examination and Diagnosis

Topic 3, accounting for 20.12% (24,991/124,211) of the discussions, focused on kidney disease tests and diagnostic content. Terms related to specific test items, such as “blood pressure,” “creatinine,” and “hematuria,” as well as test methods such as “tissue examination” and “blood test,” were particularly frequent.

The following are examples of posts using those keywords:

My blood pressure was too low during a blood test.

Which is more important, creatinine levels or the glomerular filtration rate?

In addition, discussions often included receiving a diagnosis from a physician based on test results, with terms such as “normal,” “management,” “outpatient,” “doctor,” “maintenance,” and “current” appearing frequently. Examples of those posts are shown as follows:

I came for an outpatient consultation to review my test results.

The doctor said that, as of now, my test results showed normal creatinine levels and glomerular filtration rate, but ongoing management will be necessary for maintenance.

#### Topic 4: Disease Management

Topic 4, accounting for 11.67% (14,495/124,211) of the discussions, focused on the management of kidney diseases, including exercise, water intake, and dietary management. Major keywords such as “exercise” and “weight” were prevalent in exercise management discussions such as the following:

What exercises should I do to lose weight?

Keywords such as “water” and “urine volume” were commonly observed in posts related to water intake management, such as “How much *water* do dialysis patients typically consume?”

Keywords such as “food,” “snack,” “meal,” “diet,” “eat,” “potassium,” and “control” were identified in the context of dietary management. Examples of those posts are shown as follows:

What are some high-calorie foods or snacks that don’t strain the kidneys?

Is it okay to eat snacks after dialysis?

I’m following a low-protein, low-potassium diet. What should I be cautious of when planning my meals?

#### Topic 5: Surgery for Dialysis

Topic 5, which accounted for 9.71% (12,061/124,211) of the posts, focused on dialysis and predialysis procedures. Major keywords such as “hemodialysis,” “peritoneal dialysis,” “recommendation,” and “pain” were prevalent, with many posters seeking advice on the reasons for selecting and transitioning between the 2 types of dialysis. Examples of those posts are shown as follows:

Give me a recommendation on whether I should choose hemodialysis or peritoneal dialysis.

While undergoing hemodialysis, I experienced pain as the pressure increased.

In addition, keywords such as “blood vessels,” “procedure,” “surgical site,” “anesthesia,” “surgery,” “arteriovenous fistula,” and “Seoul” frequently appeared in posts related to predialysis surgery, including arteriovenous fistula creation or placement surgery and peritoneal dialysis catheter insertion. The following are some examples:

Can you recommend a good hospital in Seoul for arteriovenous fistula surgery?

After an arteriovenous fistula surgery, my blood vessel feels enlarged, and the surgical site is swollen. Is it normal for the vessel to enlarge after the procedure?

#### Topic 6: Costs and Insurance

In topic 6, accounting for 9.03% (11,216/124,211) of the posts, the users expressed significant concerns regarding financial and insurance-related issues with respect to managing their health conditions. The major keywords were “application,” “price,” “registration,” “insurance,” and “benefits.” They appeared in sentences such as the following:

What documents do I need to prepare for an insurance application and reimbursement for costs?

How much compensation do patients undergoing dialysis receive?

I am a kidney donor and have registered for insurance. Can I claim insurance benefits?

### Distribution of Topics Across the Boards

**Figure 5:**
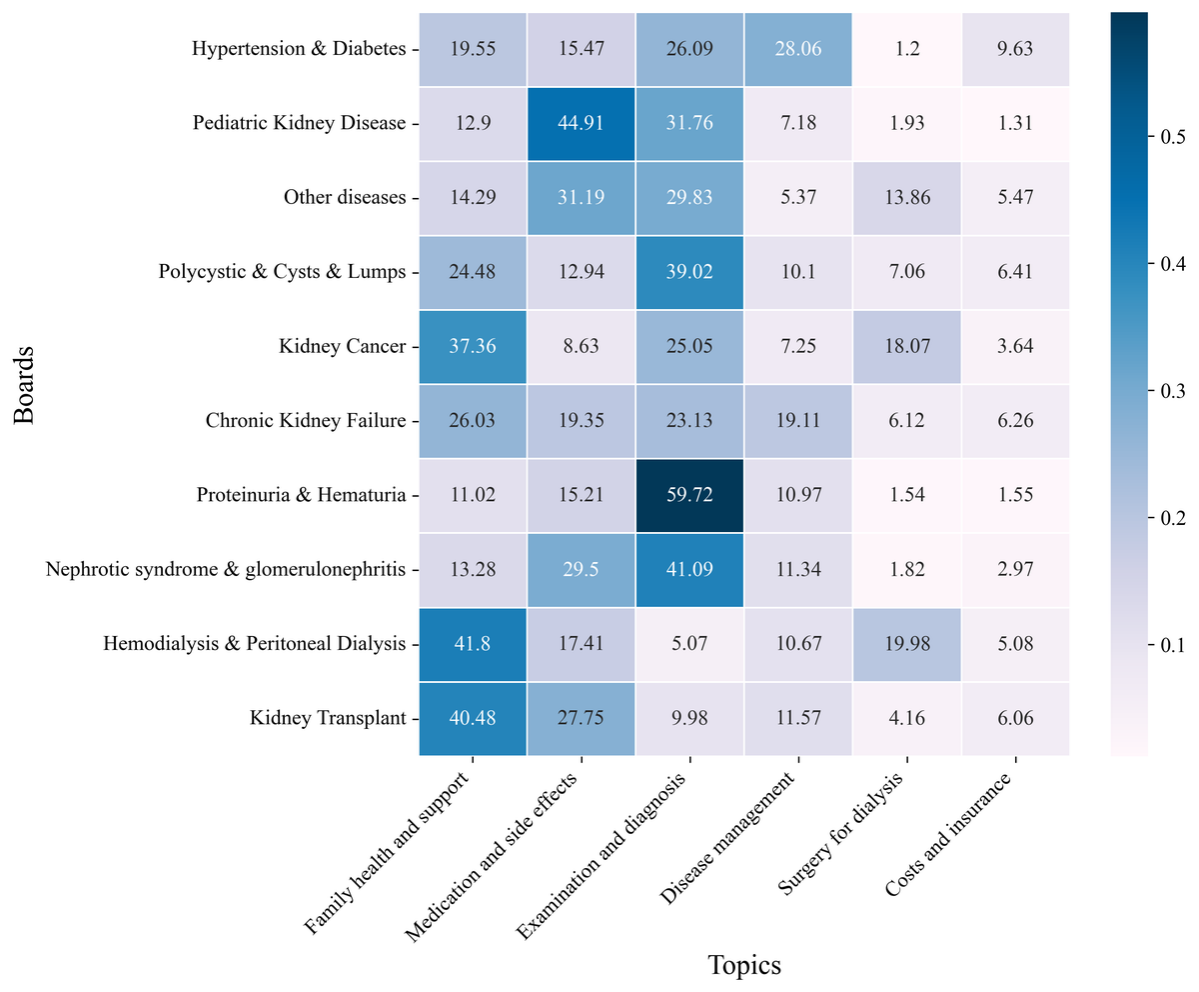
Topic distribution per board. The intensity of the colors represents the frequency of a particular topic on a board, with darker shades indicating a higher concentration of posts related to that topic. The topics are ordered from left to right in descending order of overall prevalence across all boards. The boards are arranged from bottom to top in descending order of total post count.

We analyzed the distribution of topics across different boards to gain insights into the specific focus areas in each board (Figure 5). Our analysis revealed that the Kidney Transplant board had the highest concentration of posts related to the *family health and support* topic and also showed a significant focus on the *medication and side effects* topic. Of note was that the Hemodialysis & Peritoneal Dialysis board had a higher proportion of transplant-related posts than of dialysis-specific ones. The Nephrotic Syndrome & Glomerulonephritis and Proteinuria & Hematuria boards showed a high concentration of posts related to the *examination and diagnosis* topic. The Pediatric Kidney Disease board notably focused on the *medication and side effects* topic, whereas the *disease management* topic was particularly prominent in the Hypertension & Diabetes board.

### Sentiment Analysis of the Kidney Disease Topics

**Figure 6:**
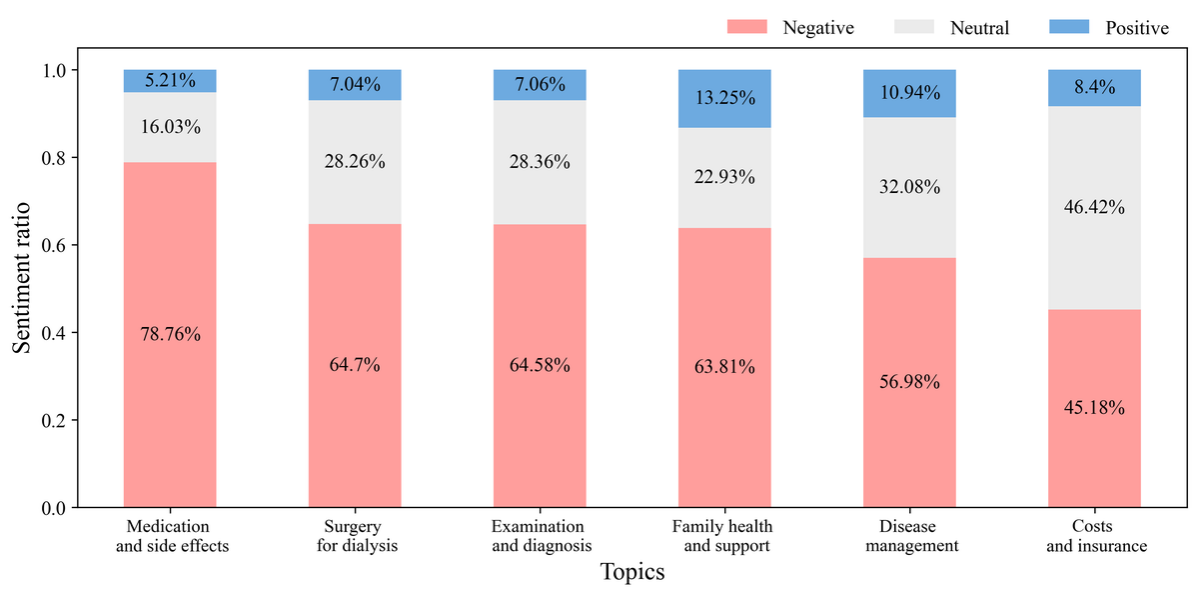
Sentiment analysis by topic—the distribution of positive, neutral, and negative sentiments across the 6 topics, demonstrating the varied emotional responses within the community discussions.

Posts on each topic cluster were analyzed to determine their sentiment, which was then categorized as positive, neutral, or negative. The results indicated that posts on the *medication and side effects* topic contained the highest proportion of negative sentiments (Figure 6). This cluster often included discussions about adverse reactions to prescribed medications, concerns about the impact of other drugs on kidney health, and general anxiety about the side effects of medications used to manage kidney disease. The following are a few posts from the community expressing these sentiments:

Since starting steroids, my eyes feel very dry, and my legs feel heavy. Is this normal?

As a polycystic kidney patient, is it safe to be prescribed medication for severe itching from a dermatologist?

While many posts described challenges, some shared positive experiences, such as temporary relief from side effects:

It was incredibly itchy and kept spreading, so I tried applying various remedies. However, the neem oil I bought from Homeplus yesterday had an immediate effect on the itching.

Similarly, the *surgery for dialysis* and *examination and diagnosis* topics mainly comprised posts with negative sentiments. These posts often discussed difficulties with dialysis, dissatisfaction with diagnostic outcomes related to kidney disease, and other related issues. The following are some examples from each topic:

I’m currently in my third year of peritoneal dialysis. I’m not anemic, but I feel extremely dizzy. Does anyone have any advice?

Last year, I was diagnosed with stage 4 polycystic kidney disease, which was tough. Now, finding out that my son has also inherited it makes it even harder.

Positive sentiments were observed when participants shared their favorable test results:

Last December’s blood test was normal, and today’s urine test also came back clean. I’m relieved but daunted by the thought of restarting my diet management.

The *family health and support* topic also comprised posts with many negative sentiments, primarily expressing worries regarding kidney transplantation among family members:

My sister is insisting on donating immediately, but I’m too worried about her health.

However, alongside these concerns, high proportions of posts about physical improvements after transplantation were also notable among the positive sentiments:

It’s been three years since my simultaneous pancreas-kidney transplant. I just tried a urine test kit, and the results are good, which makes me happy.

I’m so happy to receive such warm hearts from everyone who left comments. I sincerely thank both the patient-commenters and the patient-readers.

While the *disease management* topic also had significant negative sentiments, they were relatively fewer compared to the negative sentiments in other topics. Negative posters often expressed frustration with managing the disease or feeling overwhelmed by uncertainties in disease management:

Managing my diet is the hardest part.

I really want to drink lots of water, but it’s sad that I can’t.

In contrast, many posts inquiring about disease management strategies were classified as expressing neutral sentiments:

Is it okay to eat wild mango jelly?

Does curry have a lot of potassium?

For lunch, I had boiled squid and shiitake mushrooms fried with egg whites, enhanced by the flavorful addition of perilla oil.

In the *costs and insurance* topic, negative and neutral sentiments were expressed in similar proportions. Negative posts often focused on concerns regarding delays or cost issues in insurance paperwork, such as the following:

I applied for reimbursement of my cancer diagnosis fee, but the review process is taking too long.

I currently have workplace group supplemental insurance, but I’m worried about what will happen after retirement.

Satisfaction with the health care system was occasionally noted:

Korea’s health insurance is exceptional, covering both residential treatment and transplant surgery costs.

In contrast, neutral posts mainly shared information regarding the handling of costs or insurance, such as the following:

Today, when I visited the local district office, they provided me with an informational leaflet regarding the required documents for registering as a person with kidney disease.

If my grade 2 renal impairment progresses to grade 5, what changes will occur in my benefits?

### In-Depth Analysis of the Disease Management Topic

**Figure 7:**
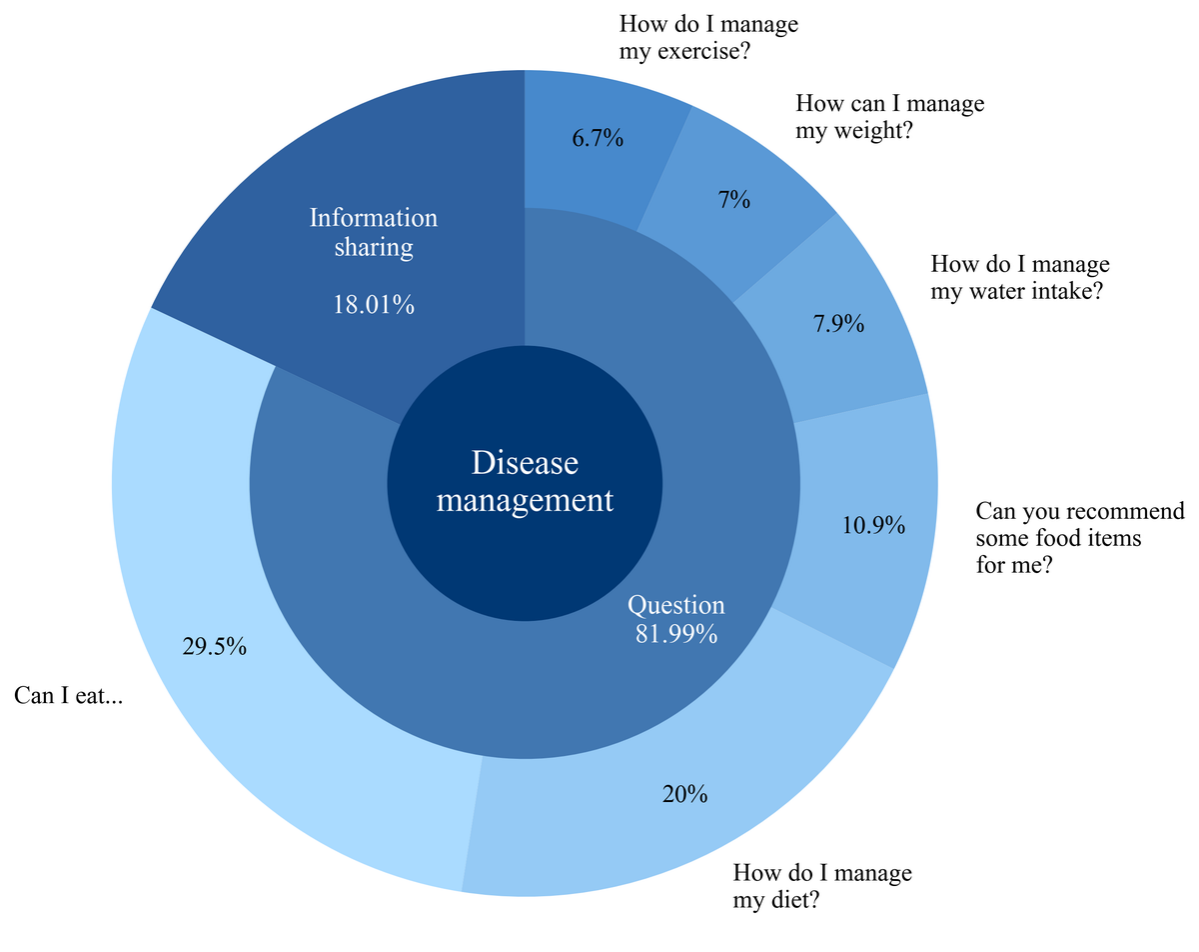
Subtopics in the disease management topic.

**Figure 8:**
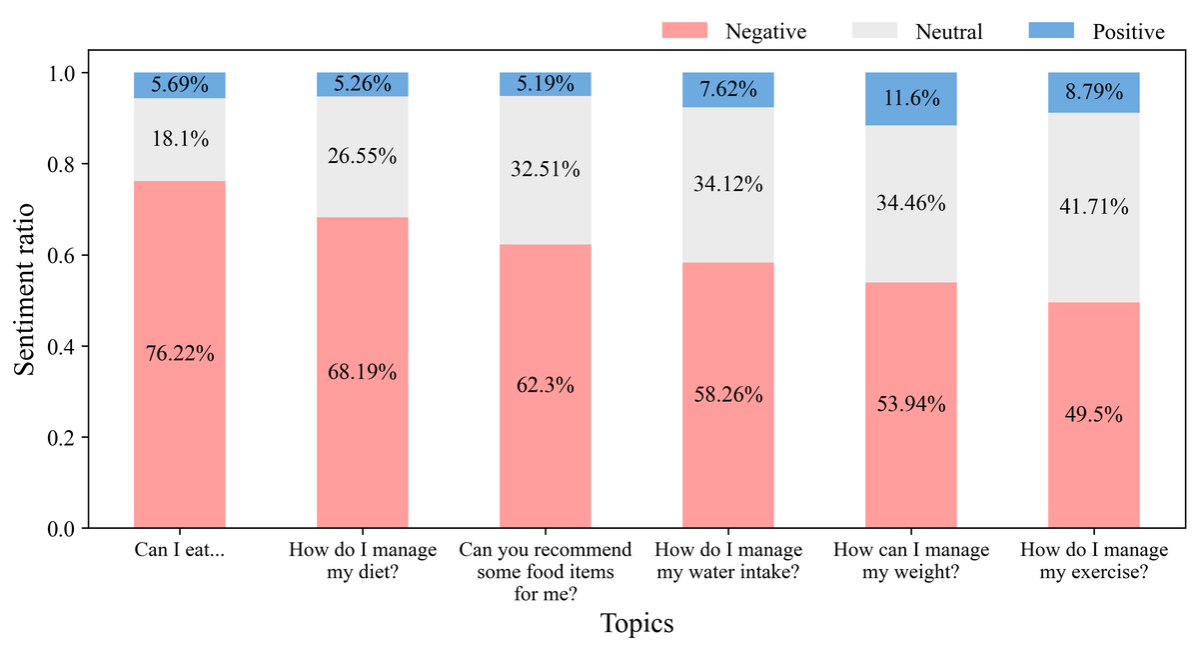
Sentiment analysis by disease management subtopic—the distribution of positive, neutral, and negative sentiments across the 6 subtopics, demonstrating the varied emotional responses within the disease management topic.

Given the complexity and importance of self-management for patients with kidney disease, we conducted an extensive analysis of the *disease management* topic. We carefully sorted the posts in this category into 2 main groups: information sharing and questions, as shown in Figure 7. We used an ELECTRA-based model to further categorize the questions into 6 specific subtopics. This process allowed us to qualitatively examine which issues were being actively discussed. We also analyzed the sentiments associated with each specific subtopic, which was important for determining which subtopics were causing more difficulties for the patients (Figure 8).

The number of posts under the *questions* category was >4 times higher than that of posts under *information sharing* (Figures 7 and 8). The *information sharing* category included valuable resources such as *Recommended information for post-transplant patients* and *Nephrotic syndrome management methods extracted from the Korean Dietetic Association*. Most posts in the *questions* category were related to dietary management (Figure 7), and these subtopics also comprised posts with more negative sentiments (Figure 8). Specifically, 29.5% (4276/14,495) of the inquiries were about the safety of specific foods, 20% (2899/14,495) sought guidance on dietary management strategies, and 10.9% (1580/14,495) requested dietary recommendations. Examples of inquiries about the safety of specific foods included the following:

Can I eat ginger if I have diabetes or kidney disease?

Are low-sodium salt and low-sodium soy sauce sold in stores okay?

Examples of inquiries seeking guidance on dietary management strategies included the following:

How should a single working person manage their weekday diet?

How should I manage my diet for proteinuria?

Examples of inquiries requesting dietary recommendations included the following:

Can you recommend low-potassium foods?

Other popular subtopics included water intake management (1145/14,495, 9%), weight management (1015/14,495, 7%), and exercise management (971/14,495, 6.7%). Examples of these questions included the following:

Is it okay to do high-intensity exercises like CrossFit if I have polycystic kidney disease?

What should I do if my weight keeps dropping?

How many liters of water do you drink per day?

## Discussion

### Principal Findings

Our study findings provide valuable insights into the concerns and challenges faced by patients with different kidney diseases and conditions. The analysis of online community discussions revealed several key points with important implications for patient care, education, and support.

#### Impact of the COVID-19 Pandemic

The analysis revealed a significant increase in community activity during the COVID-19 pandemic. This observed increase underscores the heightened health concerns and information needs of patients with CKD during this period. This finding aligns with research highlighting the increased vulnerability of patients with CKD to COVID-19 complications [50-52] and emphasizes the importance of online communities as sources of support and information during health crises.

#### Topic Distribution and Varied Information Needs Across Disease Stages

Our analysis identified six main discussion topics in the kidney disease community: (1) *family health and support*, focusing on concerns surrounding kidney donation among family members and posttransplantation management; (2) *medication and side effects*, where conversations centered on medication administration methods and the associated side effects; (3) *tests and diagnostics*, where results of blood tests and tissue examinations were predominantly discussed; (4) *disease management*, which involved advice and shared experiences related to exercise, diet, and fluid intake; (5) *surgery for dialysis*, which addressed dialysis methods and surgical procedures; and (6) *costs and insurance*, covering financial burdens related to managing kidney disease and insurance issues.

Our findings demonstrate distinct differences in patient concerns and priorities across various stages of kidney disease. Early-stage patients, as evidenced by discussions on the Nephrotic Syndrome & Glomerulonephritis and Proteinuria & Hematuria boards, primarily focused on examinations and diagnoses. These discussions often included inquiries about test results, such as creatinine levels and glomerular filtration rates, as well as clarifications on diagnostic procedures. This reflects a strong emphasis on understanding their condition and its implications. This aligns with prior work that emphasizes the importance of initial diagnostic understanding in shaping early-stage patient behaviors [53,54].

As the disease progresses, the focus shifts to managing daily life with kidney disease. On the Chronic Kidney Failure board, discussions centered on dietary restrictions, medication adherence, and emotional coping strategies. These findings align with qualitative studies that highlight the barriers to and facilitators of self-management, emphasizing that managing diet, medication regimens, and psychological well-being are critical yet challenging [55,56].

In the later stages of kidney disease, particularly for patients undergoing dialysis, discussions about transplantation became more prominent. On the Hemodialysis & Peritoneal Dialysis board, many patients compared dialysis methods and sought advice on the transplantation process. These findings underscore the transitional nature of this stage as patients undergoing dialysis often consider or prepare for transplantation. Previous research has similarly highlighted the psychological and logistical complexities associated with the transition from dialysis to transplantation [56].

For recipients of transplants, the focus shifted to challenges unique to posttransplantation life. Posts on the Kidney Transplant board were dominated by topics related to *family health and support* as well as *medication and side effects*. These discussions reflect the complex challenges faced by recipients of transplants, including adherence to strict medication regimens, management of side effects, and navigation of relationships with family members who may have been donors. This aligns with previous qualitative research highlighting the importance of social support, effective communication, and self-management strategies in facilitating successful posttransplant adaptation [56]. Posts also revealed emotional and relational challenges as recipients of transplants grappled with their roles within family dynamics and the impact of their health on loved ones.

In addition to these stage-specific concerns, our analysis highlighted challenges faced by patients with comorbidities and special populations. The Hypertension & Diabetes board prominently featured the *disease management* topic, underscoring the added complexity of simultaneously managing multiple chronic conditions. The findings emphasize the need for integrated care approaches that address the interplay between kidney disease and common comorbidities [57,58]. The Pediatric Kidney Disease board’s notable focus on the *medication and side effects* topic reflected the heightened concern about treatment impacts on children. This underscores the importance of providing tailored support and education to meet the specific needs of these populations.

By examining shifts in patient priorities and concerns, our analysis offers valuable insights into the evolving challenges faced by patients with kidney disease at different stages of their journey. These findings underscore the importance of stage-specific, patient-centered approaches in clinical care and support services.

#### Emotional Challenges and Support

The sentiment analysis revealed that discussions about medication side effects elicited the most negative emotions. The results identified particularly high levels of negative emotions in the topics of *medication and side effects* and *surgery for dialysis*. This underscores the difficulties that patients with kidney disease face regarding pain management as pain is a significant issue for these patients and significantly impacts their quality of life. Pharmacological changes associated with kidney disease increase the risk of side effects from pain medications, complicating pain management [59,60]. Specifically, >58% of patients undergoing dialysis experience pain, with 49% rating it as moderate to severe [61]. Medications typically need to be taken in the long term, causing ongoing concerns about side effects, which can lead to heightened negative emotions. Similarly, posts on the *surgery for dialysis* topic expressed negative emotions as patients began an indefinite period of dialysis.

The *family health and support* topic showed a mix of negative sentiments. Negative sentiments regarding kidney transplants and their impact on families were also prevalent, consistent with studies showing that both recipients of transplants and their families experience significant stress and concerns [62]. However, successful kidney transplants are associated with improved health outcomes and increased life satisfaction, as indicated by the relatively higher proportion of positive sentiments in posts on this subtopic [63,64]. Thus, kidney transplantation, meaning the end of long-term dialysis and dietary restrictions, which can allow patients to return to a more normal lifestyle, can increase positive sentiments.

#### Financial Concerns

The emergence of *costs and insurance* as a significant topic indicates that financial burden is a major concern for patients with kidney disease. This finding is consistent with previous research highlighting the economic challenges faced by patients with kidney disease and their families, especially those with limited financial resources [65]. The economic strain of managing CKD encompasses expenses for medical supplies, specialized diets, and transportation to medical appointments. These challenges are exacerbated by various factors, including increased costs during hospital stays for transplantation; additional expenses due to delayed graft function, which can cost an additional average of US $18,000 [66]; and the need for multiple dialysis sessions. The severity of the kidney condition typically correlates with the magnitude of the financial impact [65-67].

The prolonged nature of kidney disease often makes it challenging for patients to maintain stable employment, further contributing to their financial difficulties. The strain of managing a long-term illness can lead to interruptions in careers, thereby increasing economic hardships for both patients and their families. Many patients and caregivers report reduced work hours or job loss, leading to further financial strain [68,69]. The financial burden varies between different countries due to differences in local health care systems, yet the impact on work hours and productivity is universally challenging [70].

#### Disease Management Challenges

Consistent with previous research highlighting the role of online communities in providing a platform for individuals to share information, experiences, and support regarding health issues [71-73], our study found significant engagement in the *disease management* topic. Posts related to disease management often included sharing daily experiences and asking for advice, particularly about dietary management, which appeared more frequently compared to exercise, fluid intake, and weight management. Previous studies [58,73-75] have identified dietary management as one of the most challenging self-care tasks for patients with kidney disease. The dietary regimen for patients with kidney disease is complex and varies based on the stage of kidney disease or remaining kidney function, necessitating comprehensive management. Individual differences in taste, including a preference for salty food, pose another challenge for managing the disease.

Patients with comorbid conditions such as obesity or diabetes experience additional challenges in disease management [57,58]. Because dietary management is different for different kidney diseases, the question of whether a food item is acceptable for their condition was the most popular among the dietary management questions. This highlights the need for precise and tailored patient education regarding disease management from health care providers.

### Comparison With Prior Work

Despite the active discussions in kidney disease communities, a notable paucity of research analyzing these communities exists. To our knowledge, the only study that has previously analyzed a kidney disease community focused on the National Kidney Foundation’s online community for CKD [73]. However, this study only qualitatively analyzed approximately 20,000 posts without using topic modeling techniques. In contrast, our study used text mining methods to analyze a larger dataset, capturing more comprehensive insights into the perspectives prevalent in kidney disease communities. Another key advantage of our study was the ability to analyze posts based on disease state, which was not possible in the National Kidney Foundation’s online community due to the lack of separate boards for different disease states. The previous study did not conduct a sentiment analysis either.

Other studies exploring the challenges faced by patients with kidney disease have relied on surveys or interviews [67,76]. Analyzing online communities offers practical and voluntary insights into consumer sentiment [77], as highlighted by previous studies [78,79]. Our approach captured a more mainstream perspective due to the vast amount of user-generated content available on the web over extended periods [79]. Traditional survey methods are limited by resource constraints in analyzing data within specific spatial and temporal boundaries. In contrast, diverse individuals contribute extensive posts on web-based platforms, making text mining particularly valuable for the qualitative analysis of such large datasets.

Our findings resonate with the themes identified in previous studies. For instance, previous studies [73,76] have highlighted themes related to *managing CKD and symptoms*, *medical tests and results*, and *navigating health care and clinical needs*, which translated to *medication and side effects*, *tests and diagnostics*, and *disease management* in our study. In addition, *family health and support* and *dialysis surgery* have been similarly categorized as themes such as *considering dialysis* and *family and transplantation*, reflecting the dynamics of CKD status. *Support on the forum*, a significant theme in previous research [73], was classified in our study as *sharing information* within *disease management*, emphasizing the role of information exchange and communication in disease management.

### Limitations

While our study provides valuable insights into the discussions and challenges in online kidney disease communities, it is not without limitations.

First, the data were limited to posts from the KidneyCafe community, which may not represent all patients with kidney disease. Individuals who do not actively participate in online communities—due to factors such as digital literacy, access to technology, or personal preferences—were likely underrepresented. This limitation may particularly affect perspectives from older adults and individuals in socioeconomically disadvantaged groups. In addition, the specific context of Korean patients with kidney disease, such as differences in race, lifestyle, economic status, diet, and health care systems, may not be fully generalizable to other countries. These factors play a crucial role in shaping patient experiences and discussions. Conducting comparative analyses that use data from various web-based platforms and formal health care databases can offer a more thorough understanding of how health care systems and societal attitudes affect patient conversations. Furthermore, cross-platform comparisons would help validate our findings and reveal any potential biases specific to certain platforms in patient narratives. Expanding future studies to encompass diverse communities and geographical regions would improve the generalizability and comprehensiveness of our findings, providing deeper insights into the experiences and needs of patients with kidney disease worldwide.

Second, while the topic modeling method (LDA) was effective in extracting main topics, it does not inherently determine the optimal number of topics and may not fully capture semantic relationships between topics. The ELECTRA model, while efficient for our classification tasks, could benefit from domain-specific pretraining on medical corpora to enhance its performance on health-related text. These methodological constraints may limit the depth and precision of the topic modeling and classification results.

Third, the manual annotation process for classifying question posts related to disease management achieved substantial interrater agreement (Cohen κ coefficient of 0.77). However, the potential for bias cannot be entirely ruled out. To mitigate this, clear and detailed annotation guidelines were developed and thoroughly discussed by all annotators before the process began. Regular discussions were held to resolve disagreements and ensure consistency in interpretations, considering additional context from posts when necessary to reach consensus. While these measures contributed to the high interrater reliability observed, the limited number of annotators may have introduced some residual bias.

Fourth, the Naver CLOVA Sentiment platform’s BERT-based analysis provides significant advantages for Korean text analysis, particularly its ability to accurately interpret nuanced, context-dependent expressions in kidney disease discussions. However, this approach also has its limitations. Domain-specific lexicon-based methods, widely used in English-language sentiment analysis, provide greater transparency by enabling researchers to trace sentiment classifications back to specific words or phrases [80-82]. These methods have demonstrated effectiveness and explainability in both medical [80,81] and public health research [82], achieving meaningful results. However, their application to Korean-language analysis is constrained by the limited scale and domain-specific coverage of existing Korean sentiment lexicons [83]. This limitation highlights the trade-offs inherent in our methodology—while the BERT-based approach effectively captures contextual nuances, it is less interpretable compared to lexicon-based methods. In addition, the Naver CLOVA Sentiment platform’s computational intensity and token limit constraints may restrict its ability to process longer posts comprehensively. These choices reflect our decision to prioritize accuracy in contextual understanding, which was crucial for analyzing complex and nuanced patient discussions in Korean medical contexts.

Future research could address these limitations by incorporating data from multiple online communities, formal health care databases, and private discussions through surveys or interviews. Combining qualitative and quantitative methods, such as incorporating expert review processes or using correlated topic models, could further refine topic selection and analysis. Expanding the scope to include diverse communities from different countries would provide broader perspectives, enhancing the comprehensiveness and generalizability of the findings. These efforts would contribute to a deeper understanding of the experiences and needs of patients with kidney disease across varied contexts.

### Clinical Practice Implications

The findings of this study have important implications for clinical practice, emphasizing the crucial role that health care providers play in addressing the emotional concerns and information needs of patients with kidney disease, as is prominently reflected in web-based discussions [84].

The high prevalence of negative sentiments in posts about medication side effects and dialysis surgery underscores the need for health care providers to proactively address patients’ fears and anxieties. Strategies may include providing detailed explanations of what to expect during treatments, offering practical coping mechanisms, and regularly monitoring patients’ emotional well-being. Research indicates that such proactive support can enhance treatment adherence and improve overall clinical outcomes [85].

Our findings underscore the necessity for clearer and more consistent dietary recommendations as diet-related inquiries dominated the *disease management* topic. Dietary adherence is particularly challenging for patients with kidney disease due to the complexity of nutritional restrictions and their impact on daily life [86]. Patients often express confusion and frustration with conflicting or vague dietary advice, which hinders effective disease management. Health care providers should prioritize offering patient-specific, practical, and easy-to-understand dietary recommendations tailored to the disease stage, comorbidities, and individual preferences. Personalized dietary counseling has been shown to improve adherence and clinical outcomes [87]. Regular follow-up consultations are essential for adjusting these recommendations based on patients’ experiences and evolving health needs.

In addition, posts expressing frustration, confusion, or anxiety underscore the importance of empathetic communication and psychosocial support from health care providers. Integrating emotional support into routine care can effectively address these concerns. By fostering empathetic patient-provider relationships and offering clear, actionable guidance on disease management—especially regarding diet—health care providers can empower patients to actively participate in their care, ultimately improving their quality of life.

### Conclusions

Our study offers an in-depth analysis of web-based discussions among patients with kidney disease, highlighting key concerns and informational needs. The findings underscore the importance of online communities in providing support and education to patients and caregivers. These insights can inform health care providers and policy makers when developing targeted interventions to address the specific needs of patients with kidney disease. Precise and tailored patient education regarding disease management from health care providers is clearly needed and can be significantly enhanced through the insights gathered from online community discussions.

## Abbreviations

AKI: acute kidney injury
BERT: Bidirectional Encoder Representations From Transformers
CKD: chronic kidney disease
ELECTRA: Efficiently Learning an Encoder That Classifies Token Replacements Accurately
LDA: latent Dirichlet allocation
NLP: natural language processing

## Data Availability

The anonymized dataset and analysis scripts used in this study are available upon reasonable request to the corresponding author.
